# Biomarkers for combat-related PTSD: focus on molecular networks from high-dimensional data

**DOI:** 10.3402/ejpt.v5.23938

**Published:** 2014-08-14

**Authors:** Thomas C. Neylan, Eric E. Schadt, Rachel Yehuda

**Affiliations:** 1 Department of Psychiatry, University of California, San Francisco, CA, USA; 2 Mental Health Service, San Francisco Veterans Affairs Medical Center, San Francisco, CA, USA; 3 Department of Genetics and Genomic Sciences, Mount Sinai School of Medicine, New York, NY, USA; 4 Department of Psychiatry, James J. Peters Veterans Affairs Medical Center, Bronx, NY, USA; 5 Department of Psychiatry and Neurobiology, Mount Sinai School of Medicine, New York, NY, USA

**Keywords:** PTSD, genomics, gene expression, proteomics, Computational Biology, risk factors

## Abstract

Posttraumatic stress disorder **(**PTSD) and other deployment-related outcomes originate from a complex interplay between constellations of changes in DNA, environmental traumatic exposures, and other biological risk factors. These factors affect not only individual genes or bio-molecules but also the entire biological networks that in turn increase or decrease the risk of illness or affect illness severity. This review focuses on recent developments in the field of systems biology which use multidimensional data to discover biological networks affected by combat exposure and post-deployment disease states. By integrating large-scale, high-dimensional molecular, physiological, clinical, and behavioral data, the molecular networks that directly respond to perturbations that can lead to PTSD can be identified and causally associated with PTSD, providing a path to identify key drivers. Reprogrammed neural progenitor cells from fibroblasts from PTSD patients could be established as an in vitro assay for high throughput screening of approved drugs to determine which drugs reverse the abnormal expression of the pathogenic biomarkers or neuronal properties.

Current understanding of pathophysiology and treatment of deployment-related medical and psychiatric disorders such as PTSD is hampered by the complexity of the human system in which these syndromes are manifested, and by the lack of knowledge regarding how exposures lead to symptoms that cause deployment-related psychological injury (Yehuda, Neylan, Flory, & McFarlane, 2013). Based on available data it is likely that PTSD and other deployment-related outcomes originate from a complex interplay between constellations of changes in DNA, environmental traumatic exposures, and other biological risk factors. These factors affect not only individual genes or bio-molecules but also entire biological networks that in turn increase or decrease the risk of illness or affect illness severity. This review will focus on recent developments in the field of systems biology which use multidimensional data to discover biological networks affected by combat exposure and post-deployment disease states.

## Focus on molecular networks

**Fig. 1:**
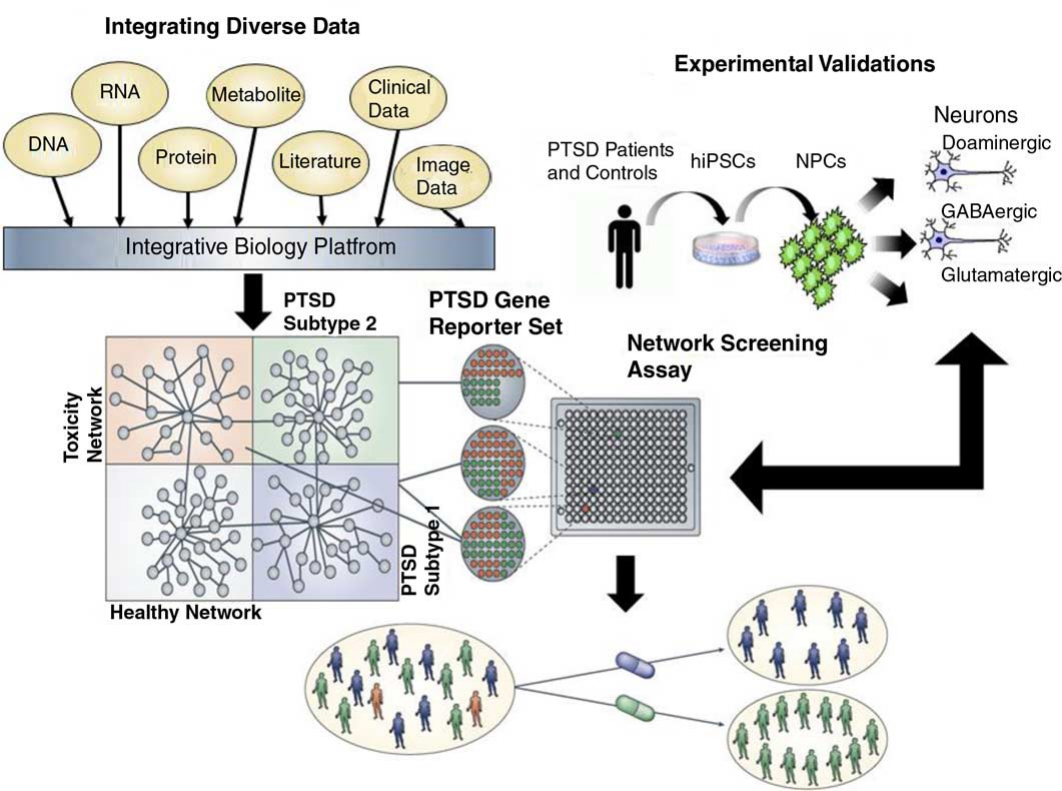
Schematic for a network approach to disease understanding and drug discovery. To understand conditions such as PTSD, we must link the molecular biology of such conditions to the pathophysiology of the condition (Schadt, 2009; Schadt et al., 2009). Integrating diverse, large-scale data provides a path to construct predictive network models of disease that in turn can inform on novel therapeutics. Here, panomic, clinical (which includes information on environmental exposures and social factors), imaging, and literature data are integrated to construct networks that inform on different subtypes of disease, healthy states, and network components associated with toxicity or other adverse events. Predictive models that define networks for a given disease subtype or toxicity can be used to construct gene expression assays that can be screened in a high throughput screening context to assess the effect any given compound has on a specific network in cells relevant to the condition under study. Screening carried out in this way can lead to the rapid identification of compounds that affect disease networks in favorable ways, while simultaneously identifying compounds that hit networks associated with toxicity or other adverse events. In this way, compounds can be identified that target specific subtypes of disease without targeting networks that can lead to toxicity or adverse events.

Our position is that PTSD symptoms can be conceptualized as emergent properties of complex molecular networks, as opposed to core biological processes associated with a disease driven by a small number of genes. Molecular networks comprise nodes, representing molecular features such as metabolite levels, protein levels, and transcript abundances and edges (Shannon et al., 2003), representing relationships between these features (Fig. 1 & Glossary). By examining molecular, cellular, and physiological features across populations of individuals under different conditions, objective, data-driven methods can be used to reconstruct the molecular networks that underlie complex phenotypes such as PTSD. This data-driven approach is a radical departure from the more hypothesis-driven approaches emphasized in previous studies that have focused on candidate genes or single biological processes and pathways involved in stress regulation. Although such studies have originated logically based on knowledge of stress responses (Yehuda & LeDoux, 2007), and have yielded important measurable differences between persons with and without PTSD, they have not explained causality of symptoms or produced reliable prognostic indicators or treatment targets. Whereas advances have been made in understanding PTSD pathophysiology, and some treatments are effective in some patients, it is still not possible to predict who will develop PTSD following exposure, who will sustain or recover from symptoms, who will respond to specific treatments, or what novel therapeutic targets may lead to more effective treatments or even prevent PTSD altogether (Yehuda et al., 2013). By integrating large-scale, high-dimensional molecular, physiological, clinical, and behavioral data, the molecular networks that directly respond to perturbations that can lead to PTSD can be identified and causally associated with PTSD, providing a path to identify key drivers of networks underlying PTSD versus passenger genes that are along for the ride. The approach set forth is one in which discovery is based on obtaining and validating multidimensional data, including genomic (genome-wide single-nucleotide polymorphisms [SNP]), epigenomic (genome-wide DNA methylation and chromatin modification), transcriptomic (gene expression, using deep sequencing [RNAseq] and RNA regulators [miRNA]), proteomic, and metabolomic data which collectively is referred to as panomics; together with careful behavioral and social phenotype data (Fig. 1).

The panomic assessment permits examination of how constellations of genetic and environmental perturbations affect the molecular states of networks and pathways that affect risk (assessed prior to exposure), prognosis (assessed post-trauma), and symptom severity. Genome-wide association studies (GWAS) are conducted to provide relevant information regarding individual responses to deployment; variations in the DNA can induce changes in molecular states driving different physiological states or symptoms. Genes may also be uniquely linked to networks that are perturbed by environmental exposures that are more proximally associated with increased disease risk (Califano, Butte, Friend, Ideker, & Schadt, 2012; Schadt, 2009; Schadt & Bjorkegren, 2012; Schadt, Friend, & Shaywitz, 2009). However, the identification of those networks requires an understanding of the molecular changes that occur once genes are activated by exposures. To fully predict susceptibility to PTSD, symptom course, and treatment response, it is necessary to identify molecular phenotypes such as RNA levels and protein states that mediate the flow of information from DNA to disease state. These provide a description of the regulatory processes and mechanisms that are likely to be causally linked with PTSD risk, onset, and progression. Because gene transcription is also regulated by epigenetic processes such as DNA methylation and chromatin marks, as well as protein and metabolite levels, all of these systems are important complements to the identification of regulatory networks. Epigenetic mechanisms are particularly important as increasing evidence implicates them in stress responses (Nestler, 2012), and increasingly, in PTSD (Yehuda & Bierer, 2009; Yehuda et al., 2013). The panomics approach is relatively new to the study of PTSD and deployment-related outcomes, but it is not new to medical science. Significant success in identification of biomarkers and treatments in other complex disorders such as irritable bowel disease (Dudley et al., 2011; Jostins et al., 2012), obesity (Cotsapas et al., 2009; Davis et al., 2012; Mehrabian et al., 2005; Yang et al., 2009; Zhao et al., 2009), diabetes (Dastani et al., 2012; Davis et al., 2012; Drake, Schadt, Davis, & Lusis, 2005; Kang et al., 2012; Keller et al., 2008; Prokunina-Olsson, Kaplan, Schadt, & Collins, 2009; Saxena et al., 2012; Schadt et al., 2003; Zhong, Beaulaurier, et al., 2010), heart disease (Derry et al., 2010; Drake, Schadt, & Lusis, 2006; Ganesh et al., 2013; Keating et al., 2008; Schwartz, Schwartz, Horvath, Schadt, & Lee, 2012; Vergeer et al., 2010), and Alzheimer's disease (Zhang et al., 2013) has been achieved using this molecular approach. Given that many of these diseases have considerable environmental antecedents—many of them highly relevant to post-deployment health outcomes—the application of these methods to the study of service persons and combat veterans with mental and physical illness is timely and appropriate.

The panomics approach has contributed to discoveries regarding illness heterogeneity and novel therapeutic targets (Holgate, 2013). In psychiatric illness, panomics methodologies have led to new models of pathophysiology, and of targeted drug effects. For instance, specific metabolomic signatures associated with depression (Kaddurah-Daouk et al., 2011), bipolar illness (Lan et al., 2009), and schizophrenia (He et al., 2012; Kaddurah-Daouk et al., 2007; Prabakaran et al., 2004) have been identified and noted to be reversed by effective pharmacotherapies. These findings have contributed to new hypotheses regarding critical illness mediators, and constitute a rationale for investigating the therapeutic potential of agents known to affect these mediators (Quinones & Kaddurah-Daouk, 2009). To date, no study of PTSD has applied a panomics methodology to examine biomarkers related to prognosis, or that change over time in association with clinical state and response to treatment.

An important first step for identifying molecular networks related to PTSD will be to conduct and replicate multiple longitudinal clinical studies of civilian and military personnel exposed to traumatic stressors. Studies from multiple animal models which capture various features of PTSD could be conducted using repeated measures and designs capturing multidimensional data. This integrative approach has the potential to establish causality and link identified networks to physiologic changes in PTSD, as has been done in other areas (Leonardson et al., 2010; Zhu et al., 2010). Although information obtained from any given system or dimension of data alone may yield a biomarker relating to some aspect of PTSD, a comprehensive understanding of this condition can only be obtained from evaluating entire biological networks that underlie PTSD (Califano et al., 2012; Schadt, 2009; Schadt & Bjorkegren, 2012; Schadt et al., 2009). This requires a multidimensional approach which examines multiple biological levels (molecular, cellular, brain circuit, organism, and interpersonal). Once we have a causal, probabilistic network framework for a disease, simulations on these networks can be carried out to predict key drivers of networks associated with disease, optimal points for therapeutic intervention, and biomarkers all of which can in turn be used to predict various outcomes including diagnosis (relative to specific molecular subtypes of disease), illness severity, persistence, recovery, recurrence, delayed onset, and response to treatment. Identifying these networks may ultimately aid in decision making (e.g., personnel placement and disposition, treatment planning) and treatment development (Califano et al., 2012; Dudley, Schadt, Sirota, Butte, & Ashley, 2010; Schadt, 2009; Schadt & Bjorkegren, 2012; Schadt et al., 2009).

### Use of animal models

In animals, it is possible to manipulate molecules and pathways to demonstrate their role in behavior, brain function, and peripheral physiology. A change in behavior resulting from a manipulation of an identified biomarker or network would constitute a strong validation of drivers of symptoms. Because no single exposure-based animal model of PTSD can adequately capture the complexity and individual variation associated with combat-related deployment, multiple models designed to reflect different aspects of PTSD (e.g., fear conditioning) are needed (Daskalakis et al., 2013). Conditional manipulations of candidate molecules with viral gene transfer and optogenetics enable examination of highly specific and nuanced alterations of biological activity (e.g., as opposed to constitutive genetic knock-outs that are non-physiologically relevant to PTSD). Viral vectors can be used in a relatively rapid and high-throughput manner, to over-express genes of interest or silence them in a site-specific, inducible fashion, with validated high-titer viruses provided to each of the sites to examine genetic manipulations across our several convergent animal models (Sparta et al., 2013). Similarly, specific brain circuits can be activated or silenced using optogenetic mechanisms to understand the neural mechanisms through which the targeted genes control complex behavior. The effects of these manipulations on animal behavior and neural molecular networks inform the relevance of blood markers to brain. It also permits identification of biological networks that must be targeted for treatment.

Biomarkers and gene networks observed in blood and multiple brain regions can be compared (initially amygdala, medial prefrontal cortex, and hippocampus, based on their extensive implication in PTSD in human and animal models, although several other brain regions can be collected and banked for future studies). This approach will allow identification of blood-based bio-markers in animals that parallel the blood-based bio-markers in humans (Yant et al., 2013). Through the knowledge of which blood-based markers best represent brain changes, we can identify the gene networks measureable in blood that are most relevant for the prevention and treatment of PTSD.

## Molecular networks and PTSD course and associated comorbidity

Biological networks associated with short-term responses to trauma exposure may also predict longer-term post-deployment symptom trajectory and long term risk for medical comorbidities. Subjects with PTSD are at substantially increased risk of cardiovascular disease, and cardiovascular and metabolic risk factors, including hypertension, dyslipidemia, obesity, diabetes (Turner, Neylan, Schiller, Li, & Cohen, 2013), dementia (Yaffe et al., 2010), and all-cause mortality. Furthermore, female veterans show increased sexually transmitted infections and gynecologic health problems (Cohen et al., 2012), asthma, emphysema, obesity, and stroke. Latent growth mixture modeling (Bonanno et al., 2012) has identified four PTSD symptom trajectories. These are 1) resilient: little to no PTSD or mental health symptoms at the current or previous wave; 2) recovered: high PTSD symptoms post-deployment that improved over time; 3) delayed: low initial PTSD symptoms that increased over time; and 4) persistent: sustained and high levels of PTSD. At present, little is known about the longitudinal course of PTSD symptoms in veterans entering into treatment. Furthermore, clinicians have no way to accurately predict which patients will recover or remain chronic over many years. High-dimensional clinical and panomic data could be used to test if molecular networks associated with persistent symptom course are also involved in the pathogenesis of associated medical comorbidities (e.g., inflammation networks).

## Approaches to inferring causality in high-dimensional data

The relationships among different clinical and molecular phenotypes and DNA genotypes can be examined to evaluate the explanatory power one trait has for another. This is useful for identifying genetic susceptibility loci, and for identifying molecular signatures associated with PTSD and related states. Networks of molecular and higher-order features (Schadt & Bjorkegren, 2012) that associate with PTSD and related phenotypes can be causally associated with these phenotypes by leveraging DNA variations, environmental conditions, and time series as systematic sources of perturbation (a necessary ingredient for causal inference) (Schadt et al., 2005; Yang et al., 2009; Zhu et al., 2007, 2008, 2012).

The basic analysis indicated above can seed the more advanced integrative methods that seek to resolve higher-order structures of many variables measured in longitudinal cohorts of subjects at high risk for trauma exposure (e.g., active duty soldiers) and determine causal relationships among them. For example, once the basic analyses confirm which gene expression traits are most strongly modulated by genetic variants, the possible molecular network structures to consider given the data can be constrained by forcing the genes affected by the causal genetic variants observed to serve as more upstream regulators of the downstream effects that result from this genetic perturbation (i.e., genes affected by a genetic variants in cis-regulatory regions that are also associated with expression traits in trans provide an obvious cause-effect relationship (Schadt et al., 2003, 2005). The construction of the molecular networks can be used to enhance the basic analyses as well, so that an iterative approach that involves performing the basic analyses in a more directed fashion based on the network architecture, that in turn has those results being used to refine the structure of the network in an iterative fashion until an optimum is achieved. Furthermore, as data continues to be generated and new data are generated (and ideally made readily available), the analyses can be updated and the models refined in a continuous fashion, not unlike the adaptive learning models that are more and more being used across a broad range of industries (quantitative finance, climatology, and high energy physics), including clinical trials.

To facilitate automated data integration, genomic, gene expression, methylation, miRNA, metabolomic, and proteomic datasets can be analyzed for links to identified upstream regulators such as transcription factors. Processed data across the levels of biological abstraction and annotation can be integrated across panomics type, species, patient populations, animal models, organs (blood, brain regions), and time point.

To more fully elucidate the complexity of PTSD using network-based approaches, network level analyses can be used to construct interaction networks in which the edges between the nodes reflect association-based relationships or direct physical interactions, and probabilistic causal networks in which the edges between the nodes are directed, reflecting statistically inferred or known causal relationships. As many experimental and computational studies have uncovered, many functional modules (sub-networks of coherent molecular activity that resides within a larger network) are highly conserved across tissues, populations, and species. Therefore, the resulting validated module-based biomarkers are more robust than the putative single gene biomarkers extracted from individual datasets. In addition to constructing different modular networks using weighted gene co-expression network analysis, candidate modules from data generated in one condition can be identified, and then these modules can be tested across other conditions to identify core network modules that are common across a disease spectrum. There are a number of advantages to this network approach: 1) The resulting modules are more compact than a disease signature and thus serve to minimize unrelated downstream signals; 2) The modules are distinct and more reasonably well-defined with respect to conditions/tissues/species that invoke them; 3) This method provides multiple robust discriminative biomarkers co-validated across experimental conditions; and 4) The modular network can reduce the overall dimensionality in the molecular space leaving open the possibility of describing it by relatively small-scale ordinary differential equation (ODE) models (Chen, Niepel, & Sorger, 2010). These advantages are particularly useful for longitudinal human observational data, treatment trials, and various animal model experiments, given the scales and complexity of data that can be generated in these settings. Combining statistical learning and mathematical modeling techniques practically reduces the complexity of high-throughput data and yet possesses powerful predictive capacity of treatment targets. The final steps in the network analyses involve key driver analysis that can integrate information from multiple network types to a final set of biomarkers.

One of the most widely used theoretical approaches and computations platforms for constructing interaction networks in which molecular features are grouped based on their degree of interconnectivity in a population of interest, is weighted gene co-expression network analysis developed by Zhang and Horvath (2005). Complementing the construction of this kind of interaction-based network is the construction of causal probabilistic networks in which DNA variation information in larger numbers samples (typically 100 or more) serves as a systematic source of variation that can be leveraged to infer causal relationships among molecular and higher-order traits. Given the random segregation of chromosomes during meiosis, DNA variations are an ideal perturbation source from which causal relationships can be inferred, similar to randomized controlled clinical trial in which randomizing patients to a given treatment arm enables causal inference (Millstein, Zhang, Zhu, & Schadt, 2009; Schadt et al., 2005). Zhang, Zhu, Schadt and others have applied these causal inference approaches to multiple studies to elucidate the complexity of living systems, especially in the context of disease (Chen et al., 2008; Emilsson et al., 2008; Schadt et al., 2003, 2005, 2008; Zhang et al., 2013; Zhong, Beaulaurier, et al., 2010; Zhong, Yang, Kaplan, Molony, & Schadt, 2010; Zhu et al., 2008, 2012). One important outcome allowed by causal network inference methods is the identification of key drivers that regulate the status of network modules of PTSD genes and are hence among the most important targets to consider for drug development.

**Fig. 2:**
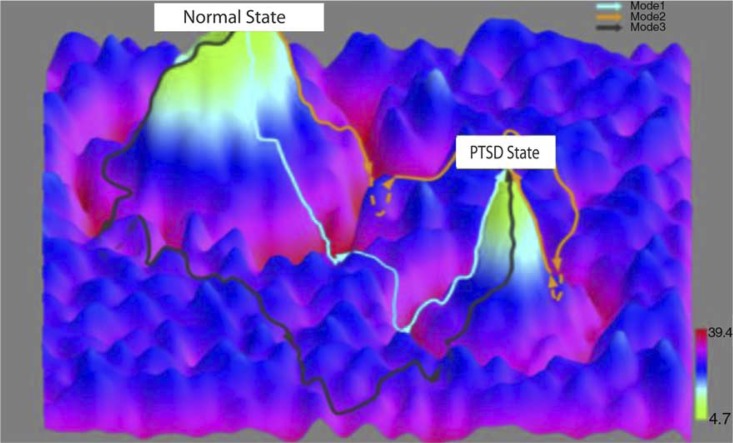
Dynamic state transitions in PTSD modeled using longitudinal panomic data. System-state trajectories between normal and PTSD-associated states. The x–y axes represent system states as defined by integrative, panomics causal networks associated with PTSD. The z axis represents potential function values that reflect the probability of being in a particular state given a network state. The contours between the normal and PTSD states represent network state transformations defined by targeting a corresponding constellation of genes using a quantitative recipe inferred from the Bayesian networks (i.e., the genes to target including the direction and level of activation or inhibition of each gene or gene product in the recipe).

Whereas interaction networks can present a global and holistic view of the interacting elements directly or indirectly involved in disease progression, probabilistic causal networks can elucidate causal relationships as well as potential mechanisms (Zhu et al., 2008, 2012). Bayesian networks represent one class of probabilistic causal modeling approaches that are in widespread use today. Bayesian networks are acyclic, directed graphs (so reflect causal relationships) in which the nodes represent molecular, cellular, clinical, or other types of variables measured in a population of interest, and the edges represent statistically inferred causal relationships between the variables (Gonik et al., 2012). Although standard Bayesian networks cannot represent feedback loops (an important construct in biology), variations of Bayesian network reconstruction algorithms such as dynamic Bayesian networks can represent such relationships (Zhu et al., 2010). Bayesian networks provide an elegant mathematical framework for integrating a diversity of data types. DNA variation, RNA variation, DNA methylation, miRNA, histone acetylation, chromatin modification, and clinical phenotype data all can be incorporated into the Bayesian network reconstruction process (Zhu et al., 2008, 2012). In general, Bayesian networks can only be solved to Markov equivalent structures (Geman & Geman, 1984), so that it is often not possible to determine the direction of a given edge unambiguously. However, the Bayesian network reconstruction algorithm can take advantage of DNA variation, genomic modifications, and environmental perturbations as a systematic source of perturbations to break this symmetry among nodes in the network that lead to Markov equivalent structures, thereby providing a way to direct edges in the network in an unambiguous fashion. State transitions between normal states and PTSD-associated states of interest derived from the causal network models can be constructed from longitudinally collected panomics and clinical data (Fig. 2).

## Multidimensional data boost power to detect genetic loci

Given the role genetic loci can play in resolving causal relationships among molecular and higher-order phenotypes, creating the most comprehensive map possible of the genetic architecture of any phenotype is advantageous on many levels. Integrative analyses can be used to substantially boost the statistical power for relating panomics data to PTSD-associated traits as well as to the DNA variants associated with them (Emilsson et al., 2008; Greenawalt et al., 2011; Zhang et al., 2013; Zhong, Beaulaurier, et al., 2010; Zhong, Yang, et al., 2010). Integrative analyses enhance the power to detect these associations by layering in additional biological data that can serve to reduce the overall dimensionality of the search space (e.g., reducing the number of SNPs that need to be tested) or by informing on putative interactions (e.g., epistasis) that may be acting together to affect certain pathways/networks driving traits of interest (Zhang, Zhu, Schadt, & Liu, 2010). The utility of integrating gene expression, expression-associated SNPs (eSNPs), and disease-associated networks with GWAS has been demonstrated to increase the power for detecting associations as well as to provide much needed functional support for identifying the most likely candidate susceptibility genes when a number of genetic loci have been identified as potential key drivers of disease (Emilsson et al., 2008; Schadt et al., 2008; Zhang et al., 2013; Zhong, Beaulaurier, et al., 2010; Zhong, Yang, et al., 2010).

**Fig. 3:**
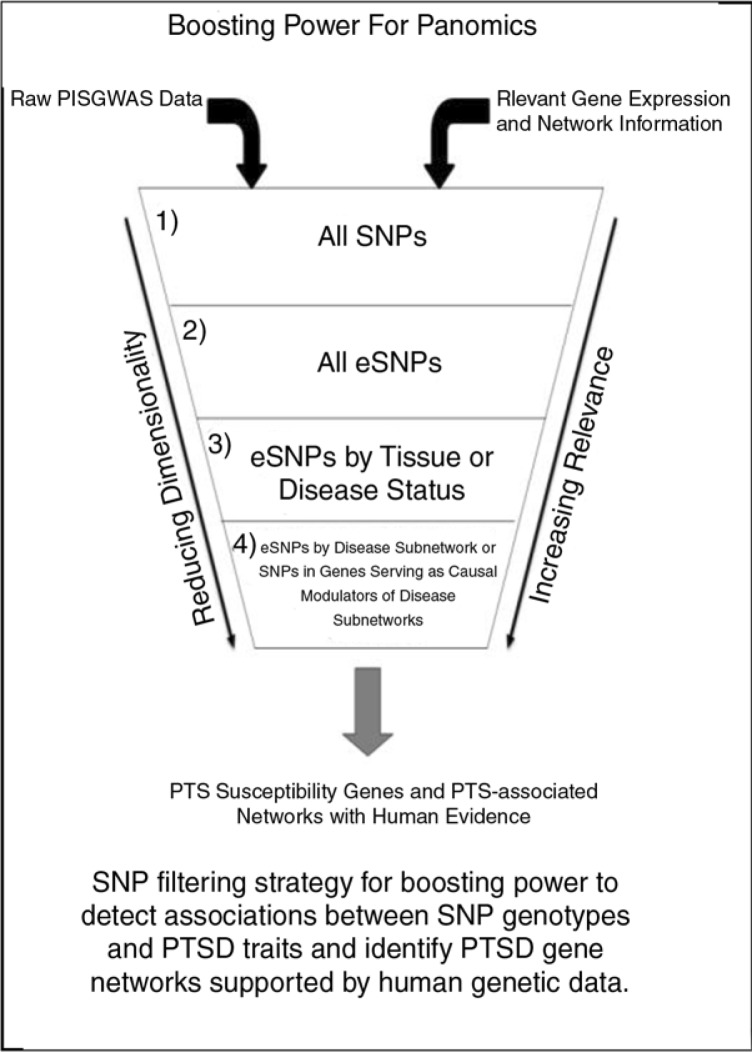
Boosting power for panomics. SNP filtering strategy for boosting power to detect associations between SNP genotypes and PTSD traits and identify PTSD gene networks supported by human genetic data.

Molecular networks operating in tissues define cellular and higher-order states that define pathophysiological states associated with disease and/or cell state. Therefore, the small nucleotide variants that associate with molecular phenotypes (e.g., expression SNPs or eSNPs) can be considered as a functionally validated set of variants that have been shown to be enriched for associating with disease traits, given they reflect perturbations in the molecular networks. Given variants like eSNPs associate with a biologically relevant phenotype like gene expression, such variants identified in longitudinal datasets can be used as a more restricted set to test for associations to PTSD (Fig. 3).

While eSNPs on their own can be a useful filter to apply in a genetic analysis, different constellations of eSNPs affect different networks that drive disease processes independently (Schadt, 2009). Organizing eSNPs into the networks they impact can dramatically improve power to identify key functional processes associated with PTSD. Clinical and panomics data can be integrated to construct weighted interaction networks and probabilistic causal gene networks, providing a framework in which to identify those sub-networks (coherent sets of genes that are interconnected in the network) associated with PTSD. Networks constructed from populations of 150–300 individuals can be sufficient to detect disease-associated networks with adequate power (Zhu et al., 2007). Whereas the power to detect associations between SNPs and traits of interest may be very low when effect sizes and/or samples sizes are smaller, the power to detect patterns of enrichment is very high, given that we can leverage constellations of eSNPs associated with a given sub-network and then test whether such SNPs are enriched for associating with the disease traits of interest (Jostins et al., 2012). There is a dramatic increase in power that can be achieved via this type of approach as indicated for many diseases, including anemia, irritable bowel syndrome, and Alzheimer's disease (Jostins et al., 2012; Zhang et al., 2013). In these studies, after identifying sub-networks associated with disease, the set of eSNPs identified over all tissues can be restricted to eSNPs that are associated with genes in the corresponding disease-associated sub-networks. This can yield enrichments over what can be expected by chance for eSNPs associated with disease including identification and validation of novel disease susceptibility genes (Greenawalt et al., 2011; Jostins et al., 2012; Zhang et al., 2013).

## Promise of human stem technology

Although animals and humans share genes, pathways, and networks, the biological context and integration of these genes with other systems may be different in different species. This can result in failures in drug development for CNS disorders. For example, many Alzheimer's disease drugs can show efficacy in animals but not translate to novel therapeutics in patients. To maximally exploit data from animal models toward understanding PTSD in humans, it is necessary to have an understanding of the genetic and molecular pathways from relevant human cells, in this case, neural cells (Fig. 1).

The ability to focus on human nerve cells relies on recently developed methods in mammals to take cells from other parts of the body (most frequently skin cells/fibroblasts) and convert them to pluripotent stem cells (called iPSC, with the “i” standing for “induced”), from which any cell can be generated, or directly into either neural progenitor cells (iNPC) or neurons (iN). The revolutionary nature of these approaches led to a Nobel Prize in October of 2012, just 6 years after Dr. Shinya Yamanaka demonstrated that with the addition of just four factors, mammalian cells can be reprogrammed into iPSCs (Takahashi & Yamanaka, 2006). Since that first paper there have been more than 1,000 papers enhancing or making use of cellular reprogramming and it has become a commonplace method. One area of active research is the re-differentiation of iPSC into neurons and other cells of the brain, because the brain is uniquely inaccessible for study on molecular and cellular levels (Marchetto, Brennand, Boyer, & Gage, 2011). Methods for generating specific types of neurons are evolving at a very rapid rate and published methods show how to bias the neurons to differentiate toward, for example, regional identities including forebrain, midbrain/hindbrain, and spinal cord (Tran, Ladran, & Brennand, 2013). With iNPC and iN, it is possible to measure the pattern of mRNA produced from each human sample using RNA-seq methodology, to carry out additional large-scale analyses of these cells, and to use iNPC and iN to test the role of genes in human neural function and to test lead compounds for drug development in these same cells. This approach also permits us to test both analytical predictions as well as biological observations from animal models in human neural cells. The utility of this approach was recently demonstrated by showing that iNPCs from patients with Alzheimer disease showed molecular alterations directly related to Alzheimer disease pathology (Qiang et al., 2011)—this is truly a human neurological disease in a dish.

One of the most encouraging examples of reprogramming in complex neuropsychiatric disorders is a recently published study by Brennand et al. (2011). In this study, fibroblasts from patients with schizophrenia were reprogrammed into iPSC and then differentiated into neurons. These neurons showed reduced neural connectivity together with decreased number of neurites, decreased levels of the synaptic protein PSD95, and reduced glutamate receptor expression. Gene expression profiles showed specific alterations in the cyclic AMP and WNT signaling pathways, and cellular and molecular abnormalities were reversed following treatment with the antipsychotic loxapine. These findings demonstrate the potential of this methodology, using skin samples of patients, to reflect complex brain functioning, including neural and synaptic development and function, together with the underlying biological pathways. These data supplement information from animal models, which result in incomplete information because the illness process in humans involves hundreds of genes and multiple pathways that differ in at least subtle ways from genes and pathways in animals. Analyzing human neural cells from PTSD subjects would yield information that could then be used to inform animal models.

## Future directions: novel treatment development

There is a unique opportunity to understand molecular changes in neural cells from individuals with and without PTSD. Reprogrammed neural progenitor cells from fibroblasts could be established as an in vitro assay for high throughput screening (e.g., to apply approved drugs from the FDA drug library to the cultured human neural cells in vitro) to determine which drugs reverse the abnormal expression of the pathogenic biomarkers or neuronal properties (connectivity, dendritic arborization, synaptic structure measured in vitro) (Fig. 1). That is, existing FDA-approved drugs could be tested for their ability to normalize molecular networks dysregulated in PTSD. This method identified a lead compound for inflammatory bowel disease (Dudley et al., 2011). Treatment development could be accelerated by understanding how different medications affect human neural cells without exposing people to medications that may not reverse PTSD pathology in the brain. Furthermore, if this approach succeeds, it may be possible to predict response to medication based on biologically validated subtypes, and by extension, individual bio-signatures along parameters that integrate genotype and changes associated with trauma exposure. Discovering the links between molecular biology and physiology will additionally provide for a rational drug discovery program and will pave the way for individualized treatment approaches (Schadt & Bjorkegren, 2012). Personalized medicine strategies require an understanding of how genetic and environmental factors perturb molecular networks that alter biological processes that lead to conditions such as PTSD (Schadt & Bjorkegren, 2012). An understanding of the molecular networks underlying PTSD will enable stratification of patient populations into subtypes and mapping of subtypes to specific treatments.

## Glossary

cis regulation: regulation of the expression of a gene by elements that are adjacent to the gene, such as promoter elements or enhancer regions of a gene

eSNPs: expression-associated single nucleotide polymorphisms-genetic variants that account for variation in expression of gene transcripts.

higher-order regulation: refers to hierarchical features involved in the regulation of biological processes. Can range across multiple levels such as molecular, cellular, circuit, organ, individual, social, and ecological strata.

key drivers: nodes in a biological networks that modulate the state of many other nodes in the network that in turn causally explain an observed physiologic phenomenon.

Markov equivalence: A mathematical term used in Bayesian statistics to describe how two sub-network structures have similar conditional probabilities with each other and as a result cannot be statistically distinguished from one another.

miRNA: microRNA. Small RNA fragments that regulate gene transcription and post-transcription modification by binding to messenger RNA.

network: a graphical structure comprised of nodes and edges in which the nodes reflect different features (variables) of interest, such as transcript levels, protein levels, protein state, and metabolite levels, and the edges reflect relationships between the nodes that are either associative (edges are undirected) or causal (edges are directed).

ODE models: Ordinary Differential Equation models- A mathematical approach for modeling biological networks which tests the rate of change of one observable biological feature as a function of one or more predictor variables.

perturbation: a force which affects the magnitude of a biological response. It is often synonymous with an external stressor; but can also be used to describe genetic variation which affects biological reactivity.

RNAseq: RNA sequencing. Refers to an advanced method for sequencing different types of RNA (e.g., messenger RNA, microRNA).

trans regulation: regulation of the expression of a gene by elements that are physically distal to the gene. Usually involves a transcript or protein that regulates a different gene.
